# Dietary Fructose Consumption and Triple-Negative Breast Cancer Incidence

**DOI:** 10.3389/fendo.2019.00367

**Published:** 2019-06-12

**Authors:** Jordan W. Strober, Matthew J. Brady

**Affiliations:** ^1^Committee on Molecular Metabolism and Nutrition, University of Chicago, Chicago, IL, United States; ^2^Department of Medicine, Section of Endocrinology, Diabetes and Metabolism, University of Chicago, Chicago, IL, United States

**Keywords:** insulin resistance, weight gain, high fructose corn syrup, breast cancer, adipose tissue

## Abstract

In the past century the western world has found a way to combat most communicative diseases; however, throughout that time the prevalence of obesity, hyperglycemia, and hyperlipidemia have drastically increased. These symptoms characterize metabolic syndrome—a non-communicable disease which has become one of the greatest health hazards of the world. During this same time period the western diet had dramatically changed. Homecooked meals have been replaced by highly-processed, calorically dense foods. This conversion to the current western diet was highlighted by the incorporation of high-fructose corn syrup (HFCS) into sweetened beverages and foods. The consumption of large amounts of dietary sugar, and fructose in particular, has been associated with an altered metabolic state, both systemically and in specific tissues. This altered metabolic state has many profound effects and is associated with many diseases, including diabetes, cardiovascular disease, and even cancer (1). Specific types of cancer, like triple-negative breast cancer (TNBC), are both responsive to dietary factors and exceptionally difficult to treat, illustrating the possibility for preventative care through dietary intervention in at risk populations. To treat these non-communicable diseases, including obesity, diabetes, and cancer, it is imperative to understand systemic and localized metabolic abnormalities that drive its progression. This review will specifically explore the links between increased dietary fructose consumption, development of metabolic disturbances and increased incidence of TNBC.

## Human Obesity

Obesity is a complex yet largely preventable disease, with overwhelmingly negative health consequences. Obesity can be defined simply by excessive fat accumulation that occurs over time when energy intake is greater than the energy demands of the body. Throughout much of the world calorically dense food is readily available and regular exercise is uncommon, which has caused a significant increase in obesity rates world-wide. Over the past 40 years global obesity has tripled and in certain populations this increase is far more pronounced. According to the World Health Organization, in 2016 39% of adults world-wide were overweight and 13% were obese. Markedly, in the United States of America 39.8% of adults 18.5% of children are obese according to data from the National Health and Nutrition Examination Survey in 2016 (2).

An individual's risk for obesity is multifaceted and is influenced by their genetic make-up, socioeconomic status (SES), activity level, sleep habits, and diet (3). For this reason, it is difficult to attribute a specific cause for the dramatic increase in obesity observed in the past four decades; however, the impact has not gone unnoticed. Medical costs attributed to obesity were approximately $40 Billion in 2006, but had increased to $150 Billion in 2014, and is estimated to approach $210 Billion in the near future (4). Much of this substantial cost is attributed to the treatment and management of comorbidities associated with obesity, including diabetes and cancer.

During times of caloric surplus, adiposity is induced as a way by which to store energy that can be utilized in caloric deficit. The ability to efficiently store energy for future fasts has been extremely advantageous throughout evolutionary history. Most simply, the body can extract energy from food by breaking chemical bonds in the carbohydrates, fats, and proteins that make up much of the diet. For this reason, prolonged consumption of high-caloric diets and minimal exercise is significantly associated with an increase in adiposity.

Due to the direct nature of fatty acids storage in adipocytes, dietary fat has been long regarded as a primary cause of obesity (5). Excessive consumption of dietary fat is strongly associated with development of diet-induced obesity, as well as induced metabolic shifts in tissues throughout the body (6). While the consumption of dietary fat has been associated with the development of obesity and metabolic syndrome for many years, the chronic overconsumption of sugar has been relatively understudied until recently.

## Type II Diabetes Mellitus

In addition to the digestion of fatty acids, simple sugars are also readily absorbed and metabolized. Glucose, fructose, and galactose are the three monosaccharides that can pass through enterocytes to enter the hepatic portal vein and be metabolized by the liver. As glucose is the main sugar substrate utilized in the human body, much of the fructose and galactose absorbed is converted into glucose or other carbon molecules used readily in cellular metabolism. A molecule of glucose can quickly be used by the cell to create energy via the glycolytic pathway and subsequent oxidative phosphorylation. These metabolic pathways catabolize glucose into carbon dioxide and water to efficiently create energy intermediates like ATP and the electron carrier NADH. The ability to rapidly create usable energy in the cell during times of great demand essential for any living organism. However, when the amount to glucose available surpasses the needs of the organism, it is vital to store the chemical energy obtained from glucose catabolism, so it can be utilized when carbohydrates are scarce.

Sugar consumption, obesity, and Type II diabetes mellitus are tightly linked. Being overweight or obese raises one's chance of developing diabetes three or seven times, respectively. Furthermore, more than 90% of people with diabetes are overweight or obese (7). The human body does an exceptional job of maintaining blood glucose levels within physiological limits—during both fed and fasted states—as deviation past the homeostatic set point in either direction can lead numerous medical complications.

Throughout fasting periods, blood glucose is maintained by breaking down energy-dense molecules stored in both adipocytes and skeletal muscle—triglyceride and glycogen, respectively. This catabolic state is mainly under the control of glucagon, which is produced by the alpha cells of the pancreas to increase glucose and fatty acid levels in the blood. Conversely, after a meal, when blood glucose levels are high, the beta cells of the pancreas release the hormone insulin, which acts to lower blood glucose levels and induce lipogenesis. With chronic over-secretion of insulin, cells throughout the body can become resistant to the actions of the hormone. As this occurs overtime, the beta cells of the pancreas will compensate by secreting increasing amounts of insulin. Eventually, with continued and chronic overeating, the beta cells will fail to produce of enough insulin to maintain normal blood glucose levels—this is the definition Type II diabetes mellitus (8). As such, elevated blood glucose levels observed in diabetes patients are treated with exogenous insulin. With the chronic overconsumption of calories causing the overstimulation of insulin and the increased rate of lipogenesis, it is easy to predict the rise of diabetes and obesity in the same patient populations. However, the metabolic changes that accompany the progression of metabolic syndrome are associated with other diseases as well. In fact, obesity and diabetes, and metabolic syndrome are all associated with a lower survival rate for those diagnosed with cancer (9–11).

## Triple Negative Breast Cancer

Cancer is characterized by uncontrolled division of abnormal cells. Over time genetic mutations of cells can accumulate and initiate tumor growth. Cancer is a heterogenous group of diseases where each is unique and labeled according to the location and genetics of the tumor. In addition to genetic abnormalities that accompany tumor growth, there is a metabolic reprogramming to support the unique needs of the actively proliferating cells. While these diseases have a wide array of symptoms, many of them exhibit a similar metabolic state of heightened glycolytic flux—known as the Warburg effect (12, 13). The catabolism of glucose is required by the transformed cell to provide energy and metabolic intermediates essential for maintaining cellular proliferation. Nearly 50 years after the altered metabolic state of tumors was recognized, the genetic basis for cancer began to be understood when Janet Rowley observed chromosomal translocation events in cells from leukemia patients (14). Since then, it has begun to be understood how the molecular biology of cancer impacts cellular metabolism and the development of the disease. This has caused drastic improvements to treatment over the past century where many cancer subtypes are now treatable.

However, breast cancer, the most frequently diagnosed female cancer, is the leading cause of cancer deaths in females, and continues to increase in incidence throughout the world (15). Breast cancers are categorized based on cellular markers reflecting available targeted therapies. TNBC is a heterogeneous set of cancers grouped by their absence of estrogen receptor (ER), progesterone receptor (PR), and human epidermal growth factor receptor 2 (HER2/neu) (16). Lacking targetable receptors for therapies, TNBC remains disproportionately more difficult to treat than other invasive breast cancers, mandating further investigation into the mechanisms driving the initiation and progression of the disease. Furthermore, due to difficulty in treating the disease, it is necessary to identify early interventions, such as diet, that may aid to reduce risk in high-risk populations.

Since Otto Warburg discovered the altered metabolic state of tumors, much effort has gone toward understanding the unique systemic and localized metabolic programming that often accompanies tumor development. Recently, the role of the mammary adipose tissue and the tumor microenvironment in the initiation and development of breast cancer has been a topic of much investigation (17–19). Adipose tissue is a well-described endocrine organ which secretes factors, including bioactive lipids, that can affect both local and systemic metabolic signaling. Specific metabolic changes in mammary adipose tissue are linked to the progression of breast cancer through the establishment of pro-tumorigenic microenvironment (20–22). Moreover, obesity and metabolic syndrome are associated with dysregulated adipocyte metabolism and alterations in the profile of secreted adipokines (23). Dietary components, as well as other stressors, are associated with alterations to the mammary adipose tissue and tumor microenvironment. Furthermore, obesity—which by itself exerts tremendous effects on adipocyte metabolism—is a significant risk factor for TNBC occurrence (24). As dietary fat has long been suspected as the primary driver of obesity, it is also hypothesized to contribute to the development of TNBC (24–26).

TNBC disproportionately effects obese, African American and lower income populations (27, 28). While the link between African Americans and TNBC has been at least partially explained by specific genetic and molecular mechanisms, further studies are needed to elucidate the remaining disparities. Interestingly, dietary fat and sugar consumption, as well as the consumption of processed foods generally high in fructose, are also increased in similar populations (29, 30). Furthermore, dietary fructose—an increasingly prominent proportion of sugar consumption—is linked with obesity and is hypothesized to upregulate pathways involved with tumor development (31–35). Obesity, which is tightly associated with the chronic overconsumption of fructose, is also one of the largest predictors of breast cancer development (36, 37).

## Fructose Consumption

Despite the connection between cancer, obesity and diabetes, for many years dietary fat consumption was reported to be the main contributor to the rising rates of obesity, and its comorbidities (38). Unfortunately, unethical practices from scientists as well as the sugar industry, obscured the metabolic consequences of high-carbohydrate diets (39). Since then, the ability of excess sugar consumption to increase lipogenesis and inducing endocrine changes has begun to be understood.

Glucose is the major for of sugar utilized throughout the body; however, fructose and galactose can also be absorbed through enterocytes lining the intestines. As fructose is absorbed through a unique channel compared to that of glucose and galactose, and insulin is only directly responsive to changes in glucose, fructose was once hypothesized to be useful in the treatment of diabetes as fructose consumption would not be predicted to aggravate hyperinsulinemic states. However, since most fructose is converted to glucose within the liver, high-fructose diets still have the ability to raise blood glucose and stimulate insulin secretion (40). Furthermore, diets with a high percentage of fructose have found to promote significant metabolic alterations (41–44).

Dietary fructose is found naturally in fruits and vegetables, as well as artificially sweetened foods, and has been found to promote metabolic changes at elevated concentrations (32). Because it is sweeter than glucose in equal concentrations, fructose has been used increasingly as an artificial sweetener. To be used more efficiently as a sweetener, high-fructose corn syrup (HFCS) began to be used in many industrial situations. To create HFCS, corn starch is broken down into glucose via an enzymatic process. Much of this glucose is subsequently converted into fructose after further processing to make a sucrose-like solution known as HFCS. While the low concentrations of fructose found in honey, fruits, and vegetables do not appear harmful, fructose consumption has increased drastically over the past few decades—paralleling the increase in obesity—since the incorporation of HFCS into processed foods was commercialized.

Since the 1950s scientists have suggested a role for sucrose (a disaccharide consisting of one glucose and one fructose molecule) in disease but the molecular and metabolic mechanisms remain unclear. Questionable scientific practices shifted the focus from sugar to fat as being a principle dietary component associated with disease (39). In the proceeding decades, the average person in the United States had shifted from eating 16–20 g of fructose to consuming 60–150 g every day (45). Furthermore, the increase in fructose consumption occurred in parallel with the increase in obesity.

## Systemic Fructose Metabolism

In addition to the metabolic reprograming stimulated by metabolic syndrome itself, the consumption of fructose is known to create distinct metabolic profiles—both systemically and in individual tissues (35, 46, 47). Once ingested, fructose is transported across the membrane of intestinal enterocytes through the GLUT5 and GLUT2 transporters (encoded by the Slc2A5 and Slc2A2 genes, respectively) into the hepatic portal vein, where it is transported to the liver (47, 48). In the liver, fructose, unlike glucose, is rapidly phosphorylated by fructokinase, bypassing hexokinase and phosphofructokinase—the rate limiting steps of glycolysis (45). As fructolysis is essentially unregulated, large quantities of fructose are quickly metabolized into lactate, glucose, and fatty acid in the liver, regardless of energy balance (49). Indeed, increased serum levels of lactate and triglyceride are observed after the ingestion of fructose (50).

Metabolic consequences of fructose consumption are even observed in children, where dietary fructose is hypothesized to dysregulate both hepatic fat metabolism and insulin kinetics (51). Interestingly, carbohydrate-responsive element binding protein (ChREBP), a central metabolic regulator that couples carbohydrate catabolism and lipogenesis, is often upregulated as a result of high fructose feeding (52). ChREBP induces lipogenesis through the upregulation of enzymes critical for *de novo* lipogenesis (DNL) (53). This stimulation of lipogenic pathways leads to the accumulation of liver fat in these children but can be readily reversed with dietary intervention. However, with increasing age, and continued consumption, the metabolic consequences only become more pronounced. When hepatic fat production, through DNL, is consistently higher than its utilization, via oxidation and export, fat will continue to accumulate in the liver, as well as ectopically. As such, both non-alcoholic fatty liver disease (NAFLD) and coronary heart disease (CHD) are strongly correlated with fructose consumption in adults (54, 55).

Furthermore, while fructose does not directly stimulate insulin release, endogenous glucose production is known to be upregulated. In addition to regulating lipogenesis, ChREBP is also a potent activator of glucose-6-phosphatase—the terminal enzyme of gluconeogenesis (53). Through these mechanisms, high-fructose feeding over time can lead to hepatic insulin resistance (56, 57). Obviously, the unregulated catabolism of fructose can strongly influence hepatic and systemic metabolism of glucose and lipid, and over time this can lead to the development of multiple diseases—including obesity, diabetes, hypertension, NAFLD, and CHD. However, in addition to these common manifestations of metabolic syndrome observed after continued high-fructose feeding, associations to other major diseases, including cancer, have been noted.

High-fructose feeding is proven to induce metabolic changes throughout the body, which are associated with the development of TNBC. While fructose does not directly induce insulin release, the endogenous glucose produced via fructolysis will stimulate insulin secretion. As insulin stimulates the cellular uptake of glucose, which can quickly be used by the cell to facilitate growth, these pathways are commonly utilized by tumors to induce proliferation. Hyperinsulinemia is closely associated with the increased expression of insulin-like growth factor-1 (IGF-1) (58). IGF-1 signaling in the mammary gland is known to induce proliferative and anti-apoptotic effects critical for tumor growth (59). Furthermore, for obese individuals with metabolic syndrome, the constant hyperinsulinemia and increasing adiposity can cause a host of effects systemically and in local tissues that are recognized to support cancer progression.

## Fructose Metabolism and the Tumor Microenvironment

**Figure 1:**
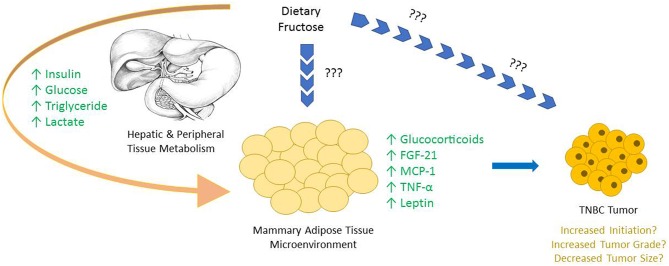
The majority of dietary fructose is catabolized by the liver, bypassing the rate limiting steps of glycolysis, creating metabolic alterations both in hepatocytes and in most peripheral tissues. Upon the consumption of fructose an increase in serum insulin, glucose, triglyceride, and lactate levels are all reported. Due to the large fructolysis capacity of hepatocytes, very little, if any fructose is seen in circulation, and the possible direct effects of fructose on peripheral tissues *in vivo* are not well-characterized. Interestingly, specific changes are observed in mammary adipose tissue and other peripheral tissues, highlighting the downstream consequences of dietary fructose metabolism. Many of these alterations are either known or hypothesized to be involved in tumor growth.

Sugar consumption has long been hypothesized to be associated with the development of certain cancers, however strong lobbying and corrupt practices funded by the Sugar Research Foundation (SRF) had impeded research in the area for many years (39, 55, 60). Notably, large quantities of dietary fructose is associated with specific metabolic changes in mammary glands that have been linked to the development of breast cancer (Figure 1) (35). Adipose tissue is a well-described endocrine organ, and the mammary adipose tissue that constitutes the tumor microenvironment of breast cancer has an integral role in the progression of the tumor.

As adipose tissue can send and coordinate signals throughout the body through the secretion of adipokines, many of these factors are hypothesized to be involved with the metabolic reprogramming of cancer cells. In fact, certain adipokines, like TNF-α, MCP-1, and Fibroblast Growth Factor (FGF)-21 have been directly implicated in the progression of breast cancer (61–63). FGF-21 regulates both systemic and localized metabolic homeostasis in many tissues. Furthermore, FGF-21 production is induced by perceived stress, as well as dietary stressors—like a high-fructose diet (42, 64, 65). In multiple cases of breast cancer, including TNBC, the receptor for FGF-21 is upregulated, suggesting a role for the growth factor in the development of the disease (66).

Another mechanism by which altered mammary gland signaling can lead to the development of breast cancer is via the activation of the glucocorticoid receptor (GR) in the tumor. Glucocorticoids are stress hormones that respond to both perceived and dietary stressors, which circulate throughout the body to influence numerous metabolic processes. Interestingly, Enhanced GR activation of mammary epithelial cells increases anti-apoptotic signaling—a key component in the development of many cancers, including TNBC (67, 68). Furthermore, diets high in fructose increase glucocorticoid production in adipocytes, which could act as a ligand for GR in the tumor epithelium. One way to stimulate GR activation is through stress signaling via the hypothalamic-pituitary-adrenal (HPA) axis. During times of stress or perceived stress, the HPA axis stimulates the secretion of cortisol in humans which binds to GR to stimulate anti-apoptotic pathways. In spontaneous rat models, chronic stress, induced through social isolation, is proven to dysregulate corticosterone—the rodent analog of cortisol—and increase tumor burden (68).

Overall the metabolic reprogramming of cancer in general leads to uncontrolled cell growth. This is achieved through stimulating growth pathways and inhibiting control offered by apoptotic signals. While many genetic factors strongly influence tumor growth, dietary factors can also influence the disease through altered systemic and microenvironmental signaling pathways, as highlighted throughout this review.

## Conclusions

Cancer, diabetes, and even obesity can be described as altered metabolic states that induce detrimental health consequences. All these conditions are greatly influenced by dietary factors that change both systemic and tissue-specific metabolic profiles. These changes, due to today's western diet high in both dietary fats and processed sugars, has led to the increased prevalence of many metabolic diseases. The molecular and metabolic links between specific nutrients in the diet such as HFCS, the development of insulin resistance and obesity as well as increased incidence of TNBC have recently been coming under greater investigation. Future work is need to understand the relative contributions of fructose consumption *per se* vs. the secondary effects of weight gain and development of insulin resistance/metabolic syndrome in the increased incidence of TNBC in at risk populations. Additionally studies, not yet conducted, into the incidence of TNBC in a subset of obese but otherwise metabolically healthy subjects with increased HFCS consumption would be a significant contribution to the field. To decrease the incidence of TNBC, more work is needed to develop dietary interventions, especially in at-risk human populations.

## Author Contributions

All authors listed have made a substantial, direct and intellectual contribution to the work, and approved it for publication.

### Conflict of Interest Statement

The authors declare that the research was conducted in the absence of any commercial or financial relationships that could be construed as a potential conflict of interest.
